# Hematological and mineral alterations associated with subclinical mastitis in dairy cattle following the foot-and-mouth disease outbreak in West Java, Indonesia

**DOI:** 10.14202/vetworld.2025.2723-2732

**Published:** 2025-09-18

**Authors:** Ronald Tarigan, Denny Widaya Lukman, Hadri Latif, Herwin Pisestyani

**Affiliations:** 1 Division of Physiology, School of Veterinary Medicine and Biomedical Sciences, IPB University, Bogor 16680, Indonesia; 2 Division of Veterinary Public Health dan Epidemiology, School of Veterinary Medicine and Biomedical Sciences, IPB University, Bogor 16680, Indonesia

**Keywords:** blood minerals, dairy cattle, hematological profile, Indonesia, somatic cell count, subclinical mastitis

## Abstract

**Background and Aim:**

Subclinical mastitis (SCM) remains a pervasive and economically significant disease in the dairy industry worldwide. In Indonesia, its prevalence has been amplified by poor management and environmental conditions, with incidence further exacerbated by the 2022–2023 foot and mouth disease (FMD) outbreak. This study aimed to investigate the hematological and blood mineral profiles of dairy cows with varying severities of SCM in West Java, thereby identifying disease-associated alterations that may improve detection and management strategies.

**Materials and Methods:**

A total of 155 blood samples and 620 milk samples were collected from Holstein-Friesian dairy cows across five high-density dairy regions in West Java between July and November 2024. Somatic cell counts (SCC) were determined using the Breed method and cows were categorized into three groups: Group A (0–100 × 10^3^ cells/mL), group B (100–400 × 10^3^ cells/mL), and group C (>400 × 10^3^ cells/mL). Hematological profiles were assessed using an automated analyzer, while serum calcium (Ca) and phosphorus (P) concentrations were measured through atomic absorption spectrophotometry. Data were analyzed using analysis of variance, Kruskal–Wallis, and Spearman’s correlation tests.

**Results:**

Elevated SCC was associated with significant increases in total leukocytes, neutrophils, and lymphocytes (p < 0.05), with a higher prevalence of leukocytosis (26.23%), neutrophilia (15.15%), and lymphocytosis (21.88%) observed in group C cows. Blood Ca levels increased significantly with SCC (r = 0.31, p < 0.01), despite overall hypocalcemia being widespread (44.78%–73.81%). Erythrocyte counts, hemoglobin, and hematocrit showed declining trends with rising SCC, though not statistically significant. No significant correlation was observed between SCC and P levels.

**Conclusion:**

Increased leukocyte, neutrophil, lymphocyte, and Ca levels are strongly linked to elevated SCC in SCM. These blood-based parameters, particularly leukocyte and Ca profiles, show promise as alternative diagnostic indicators under low-resource conditions where SCC testing is limited. This study presents one of the most comprehensive datasets on post-FMD SCM in Indonesian dairy herds, highlighting the potential for developing low-cost diagnostic markers to enhance early detection and improve herd management.

## INTRODUCTION

Mastitis is one of the most prevalent and economically significant diseases in the dairy industry worldwide. It is estimated to cause losses of up to $147/cow annually through reduced milk production, increased veterinary costs, impaired reproductive efficiency, and higher culling rates [1]. In Indonesia, the prevalence of mastitis exceeds 50% [2], largely due to inadequate management practices such as poor milking hygiene and unsanitary housing conditions. Environmental factors, particularly high humidity, further promote bacterial proliferation and increase the risk of mastitis [3]. Mastitis is an infection of the mammary gland that manifests in two forms: Clinical and subclinical. Clinical mastitis presents with visible symptoms, including udder redness, swelling, heat, pain, and abnormal milk. In contrast, subclinical mastitis (SCM) exhibits no obvious clinical signs but results in reduced milk yield. Because it can only be diagnosed through laboratory tests, SCM is considered more prevalent and economically detrimental than clinical mastitis [4].

In April 2022, the re-emergence of foot-and-mouth disease (FMD) in Indonesia affected more than 90% of dairy cattle on Java Island, resulting in a 35% decline in fresh milk production in 2023 [5]. Following the outbreak, farmers faced multiple herd health challenges, including a higher incidence of mastitis, reduced fertility, and increased zoonotic risks [5, 6]. During FMD infection, viral replication in the udder induces teat lesions that predispose cows to secondary bacterial infections and subsequent mastitis [7]. Similar trends have been reported elsewhere; for instance, within a month of an FMD outbreak in Kenya, mastitis cases increased and milk production temporarily declined [8, 9]. Beyond yield reduction, FMD also impaired milk quality and disrupted reproductive hormones, thereby compromising fertility [10]. Although no official report has documented an increase in mastitis incidence post-FMD in Indonesia, declines in milk production and quality have been recorded on dairy farms across East Java [11, 12].

Hematological and biochemical diagnostics offer valuable insights into the pathophysiology of SCM. Complete blood count (CBC) is widely used to evaluate immune responses, as mastitic cows typically show elevated white blood cell (WBC) counts, particularly neutrophils [13]. While this response is crucial for pathogen elimination, chronic or excessive activation can result in tissue damage and systemic complications. Blood mineral analysis, particularly calcium (Ca) profiling, is also relevant. Ca plays a key role in nerve transmission, muscle contraction, and immune cell function, and hypocalcemia has been identified as a major risk factor for mastitis. Low Ca levels delay teat canal closure after milking and impair immune defenses, thereby increasing susceptibility to intramammary infections [14].

Despite the high prevalence of mastitis in Indonesia and its well-documented economic impact, there is limited understanding of the systemic hematological and mineral alterations associated with SCM, particularly in regions affected by the recent FMD outbreak. Most studies in Indonesia and similar low- to-middle-income countries (LMICs) have primarily focused on farm-level risk factors, milk yield losses, or bacteriological profiles, with comparatively fewer investigations on blood-based indicators of SCM. Globally, CBC and mineral profiling are recognized as valuable diagnostic tools; however, their integration into mastitis research in tropical, resource-limited dairy systems remains scarce. Furthermore, existing reports from Indonesia do not adequately explore the interplay between FMD-related udder pathology and subsequent susceptibility to SCM. This knowledge gap is critical because conventional diagnosis based on somatic cell count (SCC) is not always accessible in smallholder farms, limiting early detection and timely intervention. Thus, identifying reliable blood-based markers for SCM under tropical, post-epidemic conditions would not only enhance diagnostic capacity but also support targeted mastitis control strategies in smallholder dairy production systems.

This study was designed to investigate the hematological and blood mineral profiles of dairy cows with SCM in West Java Province, Indonesia, an area heavily impacted by the 2022–2023 FMD outbreak. Specifically, it aimed to (i) compare leukocyte dynamics, erythrocyte indices, and serum Ca and phosphorus (P) concentrations across cows categorized by SCC levels; (ii) evaluate the associations between SCC and blood-based parameters to determine their diagnostic potential; and (iii) provide a region-specific dataset that can guide the development of alternative, low-cost diagnostic markers for SCM in smallholder tropical dairy systems. By addressing these objectives, the study seeks to contribute practical insights into mastitis monitoring and management in LMICs where advanced diagnostic facilities are limited.

## MATERIALS AND METHODS

### Ethical approval

This study was approved by the Animal Ethics Committee of the School of Veterinary Medicine and Biomedi-cal Sciences, IPB University (Approval No. 228 KEH/SKE/VII/2024). Written informed consent was obtained from local animal health offices in each city or regency. A participatory research approach was employed, involving direct engagement with farmers and verbal consent, as approved by the ethics committee.

### Study period and location

The study was conducted from July to November 2024 in five major dairy-producing regions of West Java Province: Kebon Pedes (Bogor City, 350 m above sea level); Cijeruk (Bogor Regency, 587 m); Kunak (Bogor Regency, 460 m); Pangalengan (Bandung Regency, 1,410 m); and Cigugur (Kuningan Regency, 660 m). Ambient temperatures and relative humidity during sampling were as follows: Kebon Pedes (28.7°C–32.1°C, 49.8%–53.8%), Kunak (27.6°C–31.6°C, 57.3%–74.9%), Cijeruk (26.8°C–30.3°C, 66.4%–78.2%), Pangalengan (17.0°C–23.0°C, 59.0%–94.0%), and Cigugur (21.0°C–25.0°C, 80.0%–94.0%).

### Animal selection and inclusion criteria

The study enrolled 155 purebred Holstein-Friesian cows in mid-lactation (3–6 months postpartum) and between their second and fifth lactation. A field veterinarian confirmed the health status by clinical examination. The inclusion criteria were being clinically healthy, in mid-lactation, and having no recent history of mastitis. Cows showing clinical mastitis (udder swelling, heat, pain, or abnormal milk) were excluded. Animals were selected using convenience sampling, based on availability and voluntary participation from farmers. The final sample included 22 cows from Kebon Pedes, 19 from Cijeruk, 30 from Kunak, 52 from Pangalengan, and 32 from Cigugur. Cows were grouped by mean SCC across all mammary quarters: Group A (0–100 × 10^3^ cells/mL), group B (100–400 × 10^3^ cells/mL), and group C (>400 × 10^3^ cells/mL). These thresholds followed the Indonesian standard for raw cow’s milk [15].

### Milk sample collection and SCC analysis

Teats were disinfected with 70% ethanol before collection. Approximately 10 mL of milk was sampled aseptically from each quarter into sterile tubes after discarding the first two to three streams, ensuring no contact between udder and tube edges. Samples were sealed, cooled at 4°C, and transported to the laboratory within 24 h. SCC was measured using the Breed method, a field-adaptable and cost-effective technique [16, 17]. Briefly, 0.01 mL of milk was spread on a slide (1 cm^2^ area), heat-fixed, and stained sequentially with ether alcohol (2 min), Löffler’s methylene blue (1–2 min), and 96% ethanol (1 min). Somatic cells were counted manually across 30 fields at 400× magnification (Olympus CH30RF200, Japan), and the final SCC was calculated by multiplying the mean field count by the microscopy factor (400,000).

### Blood sample collection

Twelve milliliters of blood were drawn from each cow through coccygeal venipuncture. For hematology, 2 mL was collected into K_3_EDTA-coated tubes (BD, Franklin, USA), while 10 mL was collected into clot activator tubes for mineral analysis. The serum was separated by centrifugation at 3,000 × *g* for 15 min and stored at −80°C for up to 60 days.

### Hematological analysis

CBCs were performed using an automated hematology analyzer (HM560V, Alovet, China), measuring leukocytes, erythrocytes, neutrophils, lymphocytes, monocytes, eosinophils, basophils, hemoglobin, hematocrit, mean corpuscular volume (MCV), mean corpuscular hemoglobin (MCH), mean corpuscular hemoglobin concentration (MCHC), red blood cell distribution width (RDW), platelets, and mean platelet volume (MPV). Internal quality control was ensured with B55 Normal Hematology Control (Mindray, China). Results were compared with reference ranges for mid-lactation dairy cows [18].

### Blood mineral analysis

Serum Ca and phosphorus (P) levels were determined using an atomic absorption spectrophotometer with Graphite Tube Atomizer (GTA 120; Agilent Technologies, USA). Detection limits were 0.2 μg/L for Ca and 1.0 μg/L for P. Results (mg/dL) were compared with standard dairy cow reference values [19].

### Statistical analysis

Data normality was assessed using the Shapiro–Wilk test. Normally distributed variables (MCV, MCH, platelets, eosinophils, basophils, and lymphocytes) were analyzed using one-way analysis of variance followed by Tukey’s *post hoc* test. Non-normally distributed variables were analyzed with the Kruskal–Wallis test and Dunn’s *post hoc* test. Associations between SCC and hematological/mineral parameters were tested using Spearman’s rank correlation. Effect sizes were classified as weak (ρ = 0.1–0.3), moderate (ρ = 0.3–0.5), or strong (>0.5). A p < 0.05 was considered statistically significant.

## RESULTS

### Immune cell responses

**Figure 1:**
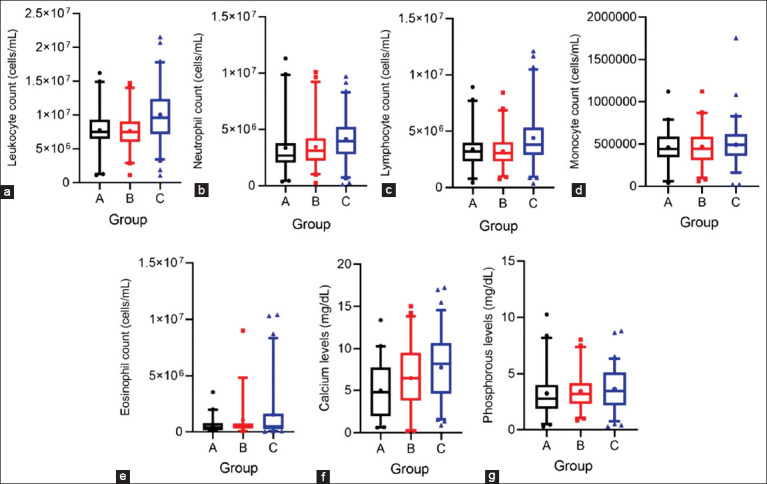
Box-and-whisker plots showing variation in leukocyte profiles and minerals in blood samples from dairy cows with different mean SCC ranges from all mammary quarters: Group A (0–100 × 10^3^ cells/mL), group B (100 × 10^3^–400 × 10^3^ cells/mL), and group C (>400 × 10^3^ cells/mL). (a) Leukocyte count (cells/mL), (b) neutrophil count (cells/mL), (c) lymphocyte count (cell/mL), (d) monocyte count (cell/mL), (e) eosinophil count (cells/mL), (f) calcium level (mg/dL), and (g) phosphorus level (mg/dL). The boxes indicate the 2^nd^ and 3^rd^ quartiles, with the median indicated by the line. The bars at the ends of the vertical lines or “whiskers” mark the 5% and 95% values. Any extreme values are indicated by solid shapes. SCC = Somatic cell count.

**Figure 2:**
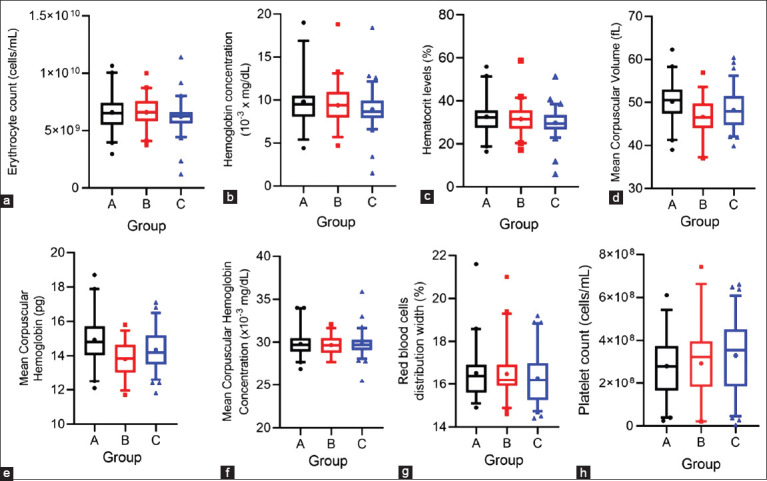
Box-and-whisker plots showing variation in erythrocyte profiles and platelets in blood samples from dairy cows with different mean SCC ranges from all mammary quarters: Group A (0–100 × 10^3^ cells/mL), group B (100 × 10^3^–400 × 10^3^ cells/mL), and group C (>400 × 10^3^ cells/mL). (a) Erythrocyte count (cells/mL), (b) hemoglobin concentration (g/dL), (c) hematocrit level (%), (d) mean corpuscular volume (fL), (e) corpuscular hemoglobin (pg), (f) corpuscular hemoglobin concentration (g/dL), (g) red blood cell distribution width (%), and (h) platelet count (cells/mL). The boxes indicate the 2^nd^ and 3^rd^ quartiles with the median indicated by the line. The bars at the ends of the vertical lines or “whiskers” mark the 5% and 95% values. Any extreme values are indicated by solid shapes. SCC = Somatic cell count.

Based on the mean SCC across all mammary quarters of 155 dairy cows, 43 cows (27.74%) were classified into group A (0–100 × 10^3^ cells/mL), 44 cows (28.38%) into group B (100–400 × 10^3^ cells/mL), and 68 cows (43.87%) into group C (>400 × 10^3^ cells/mL). The mean hematological and mineral profiles for each group are presented in Tables 1 and 2 [18], while Figures 1 and 2 illustrate the variation in selected parameters.

**Table 1:** Mean values of hematological and blood mineral profiles of dairy cows with different somatic cell count ranges.

Parameters	Unit	Reference range [18]	Group A (n = 43)	Group B (n = 44)	Group C (n = 68)
RBC	10^9^ cells/mL	5.1–7.6	6.60 ± 1.62	6.59 ± 1.28	6.23 ± 1.33
Hemoglobin	10^−3^ mg/dL	8.5–12.2	9.80 ± 2.92	9.40 ± 2.41	8.89 ± 2.10
Hematocrit	%	22–33	32.56 ± 8.03	31.49 ± 7.11	29.74 ± 5.97
RDW	%	15.5–19.7	16.53 ± 1.30	16.49 ± 1.19	16.28 ± 1.18
MCV	fL	38–50	50.16 ± 4.60^a^	46.67 ± 4.41^b^	48.27 ± 4.40^ab^
MCH	pg	14–18	14.94 ± 1.42^a^	13.81 ± 1.03^b^	14.36 ± 1.17^b^
MCHC	10^−3^ mg/dL	36–39	29.82 ± 1.57	29.67 ± 1.21	29.78 ± 1.28
WBC	10^6^ cells/mL	4.9–12.0	7.75 ± 3.59^a^	7.65 ± 2.85^a^	10.54 ± 3.90^b^
Neutrophils	10^6^ cells/mL	1.8–6.3	3.36 ± 2.42^a^	3.44 ± 1.96^ab^	4.14 ± 2.14^b^
Lymphocytes	10^6^ cells/mL	1.6–5.6	3.40 ± 1.84^ab^	3.22 ± 1.53^a^	4.47 ± 2.49^b^
Monocytes	10^6^ cells/mL	0–0.8	0.46 ± 0.21	0.47 ± 0.22	0.49 ± 0.25
Eosinophils	10^6^ cells/mL	0–0.9	0.59 ± 0.65^a^	1.05 ± 1.70^ab^	1.53 ± 2.46^b^
Basophils	10^6^ cells/mL	0–0.2	0.02 ± 0.04	0.04 ± 0.06	0.05 ± 0.06
Platelet	10^6^ cells/mL	193–637	280 ± 150	292 ± 173	330 ± 173
MPV	fL	4.6–7.4	6.74 ± 0.78	6.58 ± 1.04	6.23 ± 0.83
Calcium	mg/dL	7.6–12.4	4.97 ± 3.43^a^	6.51 ± 3.92^b^	7.77 ± 4.03^b^
Phosphorus	mg/dL	4.0–8.6	3.21 ± 2.07	3.39 ± 1.65	3.70 ± 1.85

Group A (0–100 × 10^3^ cells/mL), group B (100 × 10^3^–400 × 10^3^ cells/mL), and group C (> 400 × 10^3^ cells/mL). Values within a row followed by different superscripts^(a,b)^ are significantly different (p < 0.05). Values sharing at least one common superscript^(ab)^ are not significantly different. SCC = Somatic cell counts, RBC = Red blood cells, RDW = Red blood cell distribution width, MCV = Mean corpuscular volume, MCH = Mean corpuscular hemoglobin, MCHC = Mean corpuscular hemoglobin concentration, WBC = White blood cells, MPV = Mean platelet volume

**Table 2:** Interpretation of hematological profiles of dairy cows that are changed from the reference range among groups.

Parameters	Percentage of cows with values outside the reference ranges [18]								

	Group A		Dominant changes	Group B		Dominant changes	Group C		Dominant changes
		
	Lower (%)	Higher (%)		Lower (%)	Higher (%)		Lower (%)	Higher (%)	
RBC	17.07	24.39	↑	14.29	23.81	↑	10.61	10.61	-
Hemoglobin	29.27	14.63	↓	35.71	4.76	↓	39.39	4.55	↓
Hematocrit	7.32	36.59	↑	7.14	42.86	↑	3.03	28.79	↑
RDW	0	0	-	0	0	-	0	0	-
MCV	0	55	↑	5.56	22.22	↑	0	31.51	↑
MCH	22.50	2.50	↓	55.56	0	↓	42.47	0	↓
MCHC	100	0	↓	100	0	↓	100	0	↓
WBC	17.07	12.20	↓	11.90	9.52	↓	4.92	26.23	↑
Neutrophils	19.51	9.76	↓	16.67	7.14	↓	12.12	15.15	↑
Lymphocytes	12.20	12.20	-	11.90	7.14	↓	6.25	21.88	↑
Monocytes	0	2.44	↑	0	9.52	↑	0	4.62	↑
Eosinophils	0	17.50	↑	0	19.44	↑	0	31.51	↑
Basophils	0	0	-	0	0	-	0	0	-
Platelet	33.33	0	↓	27.78	5.56	↓	27.40	2.74	↓
MPV	0	0	-	0	0	-	0	0	-
Calcium	73.81	2.38	↓	65.12	4.65	↓	44.78	8.96	↓
Phosphorous	76.19	0	↓	74.42	0	↓	59.70	0	↓

Group A (0–100 × 10^3^ cells/mL), group B (100 × 10^3^–400 × 10^3^ cells/mL), and group C (>400 × 10^3^ cells/mL). SCC = Somatic cell counts, RBC = Red blood cells, RDW = Red blood cell distribution width, MCV = Mean corpuscular volume, MCH = Mean corpuscular hemoglobin, MCHC = Mean corpuscular hemoglobin concentration, WBC = White blood cells, MPV = Mean platelet volume. ↓ = Values below reference ranges, “- “ = Values within reference ranges , ↑: values above reference ranges

Among leukocyte indices, total leukocyte (Figure 1a) and neutrophil counts (Figure 1b) were significantly higher (p < 0.05) in group C compared with group A, although mean values remained within the reference range (Table 1). A considerable proportion of cows in group C showed leukocytosis (26.23%) and neutrophilia (15.15%), underscoring the role of neutrophils in the immune defense against intramammary infections (Table 2). Lymphocyte (Figure 1c), monocyte (Figure 1d), and eosinophil (Figure 1e) counts also increased with rising SCC, though differences were not statistically significant. Mean values remained within normal ranges, except for eosinophils, which were elevated. Notably, lymphocytosis (21.88%) and eosinophilia (31.51%) were more prevalent in group C than in groups A (12.20% and 17.50%) or B (7.14% and 19.44%). In contrast, monocyte count, basophil count, platelet count, and MPV showed no significant variation among groups and remained within normal limits (Table 1).

### Erythrocyte profiles

Several erythrocyte parameters showed declining trends with higher SCC levels. Total erythrocyte count (Figure 2a), hemoglobin concentration (Figure 2b), and hematocrit (Figure 2c) decreased progressively from group A to group C, though the differences were not statistically significant and mean values remained within the reference range (Table 1). Groups B and C, however, contained more cows with reduced hemoglobin and elevated hematocrit levels than group A (Table 2). MCV (Figure 2d) and MCH (Figure 2e) were lower in group B than in group A, with slight increases in group C. Although many cows across all groups had elevated MCV and reduced MCH relative to reference standards (Table 2), group means remained within normal ranges (Table 1). Groups B and C also had a greater proportion of cows with MCH values below the reference range compared to group A (Table 2). MCHC (Figure 2f) and RDW (Figure 2g) showed no notable differences among groups, suggesting that SCC variation did not significantly affect erythrocyte size distribution or hemoglobin concentration per cell volume.

### Blood Ca–P profiles

Serum Ca levels rose progressively with increasing SCC and differed significantly (p < 0.05) between groups B and C. Despite this trend, mean Ca concentrations across all groups were below the reference range (Table 1 and Figure 1f). Hypocalcemia was observed in 73.81% of cows in group A, 65.12% in group B, and 44.78% in group C, indicating potential Ca mobilization as part of the inflammatory response associated with SCM. Serum P levels also showed a modest upward trend with increasing SCC, though differences among groups were less pronounced (Figure 1g). Hypophosphatemia remained widespread, affecting 76.19% of cows in group A, 74.42% in group B, and 59.50% in group C (Table 2).

### Correlations between hematological parameters and SCC

Spearman’s correlation analysis (Table 3) revealed significant positive associations between SCC and total leukocyte count (r = 0.25, p < 0.05), neutrophil count (r = 0.25, p < 0.05), and lymphocyte count (r = 0.18, p < 0.05). These findings confirm that elevated SCC is associated with increased immune cell activity, particularly in neutrophils and lymphocytes, which are crucial in defending against intramammary infections. A significant positive correlation was also observed between SCC and blood Ca concentration (r = 0.31, p < 0.01), suggesting Ca mobilization during inflammation. In contrast, no significant correlations were found between SCC and erythrocyte indices (RBC, hemoglobin, hematocrit, RDW, MCV, MCH, MCHC), platelet count, MPV, or P levels.

**Table 3:** The Spearman’s correlation between mean somatic cell count and hematology parameters.

Parameter	Coefficient correlation	p-value
RBC	−0.03	0.718
Hemoglobin	−0.08	0.320
Hematocrit	−0.11	0.198
RDW	−0.05	0.204
MCV	−0.14	0.081
MCH	−0.09	0.279
MCHC	0.09	0.252
WBC	0.25	0.003
Neutrophils	0.25	0.003
Lymphocytes	0.18	0.041
Monocytes	0.06	0.491
Eosinophils	0.10	0.253
Basophils	0.09	0.280
Platelet	0.12	0.138
MPV	0.03	0.089
Calcium	0.31	0.0002
Phosphorous	0.16	0.156

RBC = Red blood cells, RDW = Red blood cell distribution width, MCV = Mean corpuscular volume, MCH = Mean corpuscular hemoglobin, MCHC = Mean corpuscular hemoglobin concentration, WBC = White blood cells, MPV = Mean platelet volume

## DISCUSSION

### Immune cell responses

The SCC in bovine milk is a widely recognized indicator of mammary gland inflammation and is strongly linked to intramammary infections. Even in the absence of clinical signs, SCM results in elevated SCC, which primarily comprises desquamated epithelial cells, macrophages, and neutrophils [20].

Unlike previous studies that evaluated hematological indices or mineral profiles separately, the present research analyzed both, offering a more comprehensive understanding of systemic changes associated with SCM. Significant positive correlations were identified between SCC, total WBC count, and neutrophils, reaffirming their role in the immune defense against mastitis pathogens. These findings are consistent with earlier reports showing elevated WBC and neutrophil counts in cows with SCM [21–23]. During inflammation, increased vascular permeability allows plasma proteins and leukocytes to migrate into infected tissues. The elevated WBC and neutrophil counts observed here likely reflect intensified immunological demand within the mammary gland. Furthermore, bacterial presence has been associated with total SCC and with proportions of leukocyte subtypes in milk [24].

In addition to leukocytes and neutrophils, SCC also correlated positively with lymphocyte counts. This contrasts with previous studies by Alhussien *et al*. [25] and Sarvesha *et al*. [26], which reported elevated granulocyte and total leukocyte counts alongside decreased lymphocyte levels in SCM. Leukocyte distribution typically shifts during the acute phase of mastitis: Neutrophils dominate in the early stages, whereas lymphocytes initially decline. However, lymphocytes may become predominant in blood and milk several days after infection [27]. In this study, lymphopenia was more frequent in cows with moderate SCC levels, suggesting acute responses, while lymphocytosis was more common in those with high SCC, suggesting chronic immune activation. Stress responses may further influence lymphocyte counts, as elevated cortisol can reduce circulating lymphocytes, particularly in stress-prone dairy cows [28].

### Erythrocyte profiles

Erythrocyte indices in this study demonstrated a declining trend in RBC count, hemoglobin concentration, and hematocrit with increasing SCC, though these changes were not statistically significant. No significant correlations were observed between these parameters and SCC. Other studies, however, have reported significant decreases in RBC, hemoglobin, and hematocrit in SCM cows [23, 29]. Cytokine activation during inflammation stimulates ion sequestration, reducing hemoglobin synthesis and contributing to hypochromic RBCs. This mechanism represents a host defense strategy to restrict iron availability to pathogens, as iron is vital for microbial growth [30]. In this study, hypochromia was reflected by numerous samples with low MCH and MCHC values.

Interestingly, a higher proportion of cows showed polycythemia, with RBC increases ranging from 10.61% to 24.39%, compared with anemia rates of 10.61%–17.07%. Polycythemia may be absolute or relative. Absolute polycythemia arises from elevated erythropoietin production, often in response to hypoxic conditions at high altitudes. This was supported by the high proportion of cows with macrocytosis (MCV elevation in 22.22%–55.00% of samples), consistent with reports of immature RBCs in high-altitude cattle [31]. Four of the five sampling sites in this study were located at high altitudes. Relative polycythemia, on the other hand, results from reduced plasma volume, commonly due to dehydration. The elevated hematocrit values in 28.79%–42.86% of cows indicate dehydration as a contributing factor.

### Blood Ca–P profiles

Serum Ca concentrations were positively correlated with SCC, echoing findings from previous studies by Lakshmi *et al*. [29], and Singh *et al*. [32]. Increased Ca levels in mastitic cows may result from reduced milk yield, thereby decreasing Ca excretion through milk. Despite this trend, hypocalcemia remained prevalent, affecting 44.78%–73.81% of cows. Hypocalcemia has been closely linked to mastitis risk, as it impairs teat sphincter contraction, prolongs teat canal openness after milking, and increases vulnerability to bacterial invasion [14]. In addition, Ca deficiency reduces neutrophil function and weakens immune defenses, particularly during early lactation [14].

Serum P levels also showed a slight upward trend with SCC, although correlations were not statistically significant. Hypophosphatemia was highly prevalent, affecting 59.70%–76.19% of cows. Because Ca and P homeostasis are interconnected, hypocalcemia stimulates parathyroid hormone (PTH) secretion, which promotes bone resorption and renal Ca reabsorption. Simultaneously, PTH enhances renal P excretion, thereby contributing to hypophosphatemia.

### Study limitations and future directions

This study demonstrates that leukocyte profiles and serum Ca levels may serve as practical biomarkers for the early detection of SCM in resource-limited settings where SCC diagnostics are not routinely available. Nevertheless, several limitations must be acknowledged. First, potential confounding factors such as altitude, nutritional status, and hydration were not fully controlled. Second, the lack of pathogen identification prevented differentiation of hematological and mineral changes by specific causative agents. Third, the cross-sectional design limited the assessment of temporal changes in hematological and mineral parameters during disease progression.

Future research should employ longitudinal study designs to monitor changes over time and validate the use of blood-based biomarkers as reliable alternatives to SCC for diagnosing SCM. Integrating pathogen profiling and environmental factors would further strengthen diagnostic accuracy and provide deeper insights into mastitis pathophysiology.

## CONCLUSION

This study demonstrated that SCM in dairy cattle is associated with distinct hematological and mineral alterations that correlate with SCC. Elevated SCC was significantly associated with increased leukocyte, neutrophil, and lymphocyte counts, as well as higher serum Ca concentrations, while erythrocyte indices showed declining but non-significant trends. Hypocalcemia and hypophosphatemia were also widespread, indicating systemic metabolic imbalances accompanying SCM. These findings suggest that leukocyte profiles and Ca dynamics could serve as alternative or complementary diagnostic markers for detecting SCM, particularly in smallholder settings where SCC-based diagnostics may not be routinely available.

From a practical perspective, the use of blood-based parameters such as leukocyte counts and Ca levels offers a low-cost and accessible diagnostic approach for early identification of SCM in resource-limited dairy systems. This is especially relevant in Indonesia and other tropical LMICs, where high mastitis prevalence is compounded by limited access to laboratory infrastructure. The identification of these parameters as potential indicators can enhance on-farm mastitis management, improve milk productivity, and reduce economic losses associated with undetected infections.

The strength of this study lies in its integration of hematological and mineral profiles with SCC data, providing a comprehensive assessment of systemic changes in cows affected by SCM. In addition, the study was conducted in West Java shortly after the 2022–2023 FMD outbreak, offering unique insights into mastitis-related blood profiles under post-epidemic and tropical field conditions.

In conclusion, leukocyte profiles and serum Ca levels represent promising diagnostic markers for SCM detection in dairy cattle, particularly under low-input farming systems. Future longitudinal studies should validate these findings across different regions and production systems and explore their utility in multiparametric diagnostic models. By combining SCC with blood-based biomarkers, more reliable and accessible diagnostic tools can be developed, ultimately supporting improved herd health, milk quality, and economic sustainability in the dairy sector.

## AUTHORS’ CONTRIBUTIONS

RT, DWL, HL, and HP: Conceptualized the study. DWL, HL, and HP: Validated, investigated, and supervised the study. RT and HP: Data collection, analysis, and interpretation. RT: Drafted the manuscript. All authors have read and approved the final manuscript.
